# Updates on Palliative Medicine in the COVID-19 Era

**DOI:** 10.3390/jcm11020315

**Published:** 2022-01-09

**Authors:** Giustino Varrassi, Martina Rekatsina

**Affiliations:** 1 Paolo Procacci Foundation, 00193 Roma, Italy; 2 Mid and South Essex University Hospitals Group, Orsett Hospital, Grays RM16 3EU, UK; mrekatsina@gmail.com

The advances in knowledge in the field of pain medicine in the last half century have recently been reported from both the scientific and the social points of view [1,2,3]. The impact of pain on quality of life and healthcare utilization is highly significant [4]. Palliative care is frequently assimilated with pain medicine, even if pain management is only one important aspect of palliative management. Palliative medicine is a medical specialty dedicated to providing relief from the stress, burden, and symptoms of an illness for patients nearing the end of life. Its goal is to provide patients with better medical management, reduce their suffering, and enhance their quality of life. In addition, palliative care should support patients’ families, relieving their psychological and physical burden to assist a terminal patient, with all the associated consequences that this entails.

Since early 2020, COVID-19 imposed changes to the many aspects of the global healthcare system [5,6,7], including pain management in primary [8] and secondary care, as well as palliative medicine [9]. Palliative patients are a vulnerable population and like other populations with a higher existing pain burden, multiple comorbidities such as cardiovascular and pulmonary disease [10], severe pre-existing neurological conditions [11,12], or dementia [13,14], they have a higher incidence of COVID-19 infections [15,16]. A complete change in healthcare access [17] due to reallocation of resources and redeployment, as well as the impact of the pandemic on the mental health of caregivers and health workers, is further challenging the teams that are already struggling to provide adequate care [18]. Additionally, we are starting to learn about the new needs of the COVID-19 palliative care patients, such as managing long-COVID-19 symptoms and breathlessness [10,19]. Moreover, palliation is a paradigm shift for healthcare professionals who are trained to use sometimes heroic and extreme measures to prolong life. In palliative care, death is no longer the enemy to be conquered at all costs but rather the inevitable result of the course of disease. This paradigm shift can be unsettling to healthcare workers and may pose burdens on the system.

Palliative medicine has been significantly affected by necessary pandemic-related adaptations in the relationship between the clinician and the patient/caregiver or family. These changes have mainly played out in the way of the patient/caregiver or family share information with the clinical team and how limited healthcare resources are utilized [20]. These new rules require social distancing and the use of personal protective equipment, both of which are formidable barriers to communication, empathy, and emotional support, which are essential elements of palliative medicine [20]. The role of telemedicine and eHealth, which has expanded dramatically in the pandemic, has not served the palliative population well, where patients need individualized support more than remote access to information. In addition, palliative patients may have limited access to or interest in technological tools [17]. Social media acts as a dipole and introduces either positive or negative information, guiding people to trusted resources or advancing misinformation [21]. In terms of peer support, social media may play a positive role, and psychological first aid is one of the advantages of social media [22].

Advanced care planning is another significant change in palliative care during COVID-19. Such planning is important in order to avoid unwanted hospitalizations and intensive care treatment. Nursing home patients and their families are involved in making decisions about the possibilities of COVID-19 infection during hospitalization. Advance directives can also help in the event that a palliative care patient requires COVID-19 hospitalization or intensive care treatment in a time when resources are scarce [15]. In the palliative care population, advanced care planning should include how to manage dyspnea, how clinicians and patients as well as family communicate, and how to support the palliative patient with a serious COVID-19 infection in terms of providing spiritual and psychosocial support. Palliative care must also embrace the patient’s family and friends and offer emotional support during their loved one’s illness and bereavement care and grief counseling when the patient dies [16].

During the pandemic, as in other challenging situations, speculation and rumors regarding treatments, medications, and vaccinations can have a significant impact on not only care, but also the confidence of patients and their families in the healthcare system. Early in the pandemic, the unfounded speculation that taking nonsteroidal anti-inflammatory drugs could be linked to severe forms of COVID-19 in young and healthy subjects might had a significant impact on pain management [23]. Luckily, it was immediately and successfully opposed by the healthcare community [24,25]. Other misinformation included the suggestion to avoid ACE2-increasing drugs for patients with cardiac diseases, hypertension, or diabetes [26]. This also created a tsunami of responses by the health community [24,27,28,29] and forced the initial authors to review and eventually withdraw their overinterpreted hypothesis [30].

Today, two years after the start of the pandemic, the situation has not changed much, as new variants of the virus arrive, usually with initial unknown severity of disease and consequences to patients and healthcare systems [31,32], but with a lot of demands by the media for ready answers and instant solutions. A recent multinational survey on changes in palliative care services during the COVID-19 pandemic showed a significant increase in activity in palliation and hospice, making it clear that the need is expanding [33]. The ongoing COVID-19 pandemic will continue to pose a considerable challenge for palliative patients and their caregivers and personnel. Among these challenges are the constant changes in healthcare provisions, new variants and the resulting misinformation or inadequate information about them, burnt-out healthcare workers, scarce resources, and the influx of complex medical cases in an already overstressed healthcare system. Within this whirlwind of challenges, palliative patients need more than just professional compassion; they need personalized care strategies that must be carried out even in lockdown. They need real-world approaches that recognize that they are a different population from others with acute infection. It is sometimes difficult to prioritize palliative care in these dark days of COVID-19, but these patients represent an important and under-served population who deserve our highest levels of attention.

Without a doubt, it is the right time to increase scientific interest in palliation as a medical specialty. In modern medicine, palliative care patients deserve something different from just the “empathy” proposed by past generations. They need a scientific approach, and this Special Issue of Journal of Clinical Medicine would be the right arena in which to help shape a modern, scientific, personalized approach to palliation, a field that has been neglected and misunderstood far too long.
